# The association between perceived relationship discord at childbirth and parental postpartum depressive symptoms: a comparison of mothers and fathers in Sweden

**DOI:** 10.3109/03009734.2012.684805

**Published:** 2012-10-30

**Authors:** Birgitta Kerstis, Gabriella Engström, Kristina Sundquist, Margareta Widarsson, Andreas Rosenblad

**Affiliations:** ^1^Centre for Clinical Research, Uppsala University, Central Hospital, Västerås, Sweden; ^2^School of Health, Care and Social Welfare, Mälardalen University, Eskilstuna, Sweden; ^3^Primary Health Care Research, Clinical Research Centre, Lund University, Malmö, Sweden

**Keywords:** Depression postpartum, family, family relations, fathers, mothers

## Abstract

**Aim.:**

To examine whether mothers' and fathers' levels of perceived relationship discord at childbirth were associated with postpartum depressive symptoms when the child was 3 months old. Another aim was to examine parents' levels of self-reported depressive symptoms. The hypothesis was that parents with high levels of perceived relationship discord have higher levels of postpartum depressive symptoms than parents with low levels of perceived relationship discord.

**Method.:**

One week after childbirth, 305 couples' perceived level of relationship discord was measured using the Dyadic Consensus Subscale (DCS) of the Dyadic Adjustment Scale (DAS). At 3 months postpartum, the same couples answered the Edinburgh Postnatal Depression Scale (EPDS) questionnaire. The relations between perceived level of relationship discord and postpartum depressive symptoms were analysed using standard non-parametric statistical methods.

**Results.:**

The mothers and fathers partly differed regarding which areas of their relationship they perceived that they disagreed with their partners about. Furthermore, 16.5% of the mothers and 8.7% of the fathers reported postpartum depressive symptoms, and there was a moderate level of correlation between the DCS and EPDS scores.

**Conclusion.:**

These results may be useful for professionals in antenatal care and child health centres as well as for family caregivers who need to be aware that mothers and fathers may have different views on relationship discord and of the high level of depressive symptoms in recent parents. Further research is needed to examine perceived relationship discord and the development of depressive symptoms postpartum over a longer term.

## Introduction

The birth of a child makes life change for an individual and her/his relationship. Shortly after the birth of a child, many parents experience feelings of emotional distress. For some, these feelings grow worse and they develop postpartum depressive symptoms (1). Several studies have described an association between marital discord and depression (2,3). The prevalence of postpartum depressive symptoms among women varies between studies; from about 10% to >20% (3–5). Postpartum depressive symptoms among men have been less well studied, but an estimate sets the prevalence at about 10% (6). In Sweden, the prevalence of depressive symptoms among women is 12.5% at 8 weeks and 8.3% at 12 weeks postpartum (7). With such a high prevalence, antenatal care, which is responsible for the postpartum follow-up of new mothers, and child health centres (CHC) will likely meet both women and men with postpartum depressive symptoms. What is lacking is a study examining parents' perceived levels of relationship discord in relation to postpartum depressive symptoms.

Developmentally, there is a risk for a child when one parent is depressed, and if both parents are depressed the risk increases even more (8). Postpartum depression aggravates a parent's ability to become involved with a child and may even lead to abuse (9). Maternal postpartum depression negatively affects mother–child bonding (10) and mother–infant interaction (11). Paternal postpartum depression is associated with later disorders in children, especially regarding the behavioural development of sons (12,13). Parental depression in early parenthood, investigated when the children were 24 months old, has been found to have a negative impact on parent-to-child reading frequency, and children of depressed fathers have a higher risk of limitations in their expressive vocabulary (14). The negative consequences of postpartum depressive symptoms make it important to identify depressive symptoms early, in order to prevent and minimize potentially harmful effects on the infant, as well as on the depressed parent (15).

Several studies have found an association between maternal and paternal postpartum depression (16–18). If one of the parents suffers from depression, the other may also develop it (19). The strongest predictor of paternal depression during the postpartum period is maternal depression (8). Low levels of social support and a previous history of depression are associated with postpartum depressive symptoms among both mothers (3) and fathers (20). For mothers, the key domains for acquiring postpartum depression are a past history of depression (whether postnatal or otherwise), a history of abuse, and personality style (21). Cox et al. found no difference in the point prevalence of depression for mothers measured 6 months after childbirth (9.1%) compared to a group of control women who were neither pregnant nor had given birth in the previous 12 months (8.2%), but a 3-fold higher rate of onset of depression was found within 5 weeks of childbirth (22). Pregnant women are less likely than non-pregnant women of child-bearing age to seek treatment for mental disorder (23). If a mother or father suffers from postpartum depressive symptoms it can affect not only the person him-/herself but also their relationship and their child (12,16).

Transition to parenthood may involve a decline in marital satisfaction for both women and men (24). Thus, it could be expected that low levels of parental relationship quality might be associated with postpartum depressive symptoms. Despite the fact that postpartum depressive symptoms are common among both women and men, research concerning the association between postpartum depressive symptoms and relationship discord is scarce. The study by Salmela-Aro showed that a high level of postpartum depressive symptoms is associated with a low level of relationship satisfaction (25). However, to the best of our knowledge, no previous research has studied the association between parents' perceived level of relationship discord in specific areas (e.g. recreational activities or household tasks) and postpartum depressive symptoms. The aim of the present study was to examine whether mothers' and fathers' levels of perceived relationship discord at childbirth were associated with postpartum depressive symptoms when the child was 3 months old. Another aim was to examine parents' levels of self-reported depressive symptoms. The hypothesis was that parents with high levels of perceived relationship discord had higher levels of postpartum depressive symptoms than parents with low levels of perceived relationship discord.

## Materials and methods

The present study is part of an extensive cohort study which aims to discover if there are differences in mothers' and fathers' views on children and the family, and whether the father's involvement can be beneficial for a child's physical and psycho-social well-being. Structured questionnaires were given to mothers and fathers during the first visit to the CHC (baseline) and at 3 months postpartum. At baseline, the mothers' and fathers' perceived levels of relationship discord were measured using the Dyadic Consensus Subscale (DCS) of the Dyadic Adjustment Scale (DAS) (26). Three months postpartum, the parents' self-reported depressive symptoms were measured using the Edinburgh Postnatal Depression Scale (EPDS) (27). Further, the mothers and fathers answered demographic questions in the baseline questionnaire.

### Data collection procedures

Swedish-speaking parents of children born through the years 2004–2006 in the northern part of the county of Västmanland, Sweden, were asked by the CHC nurses to participate in the study. The parents were recruited consecutively, regardless of whether it was their first child or whether they were already parents. The baseline questionnaire was answered by 100% of the mothers and 98% of the fathers. The aim was to recruit 400 couples, since a power calculation before the study concluded that a response rate of 67% from 380 couples was needed. The aim was well achieved when 401 couples were included.

At baseline the CHC nurses gave the parents oral and written information about the project. Thorough instructions emphasized that it was important that the questionnaires were filled in separately by each parent. A cover letter explaining the aim of the study and information about how to contact the research team was attached to each questionnaire. The baseline questionnaire was answered at the CHC, while the follow-up questionnaire was sent to the parents' home by mail when the child was 3 months old. This latter questionnaire was returned by mail in a prepaid envelope. A postal reminder, including an identical questionnaire, was sent once to those who did not reply within 3 weeks. A second reminder occurred by telephone 5 weeks later. All questionnaires were decoded and depersonalized before analysis.

**Figure 1.**
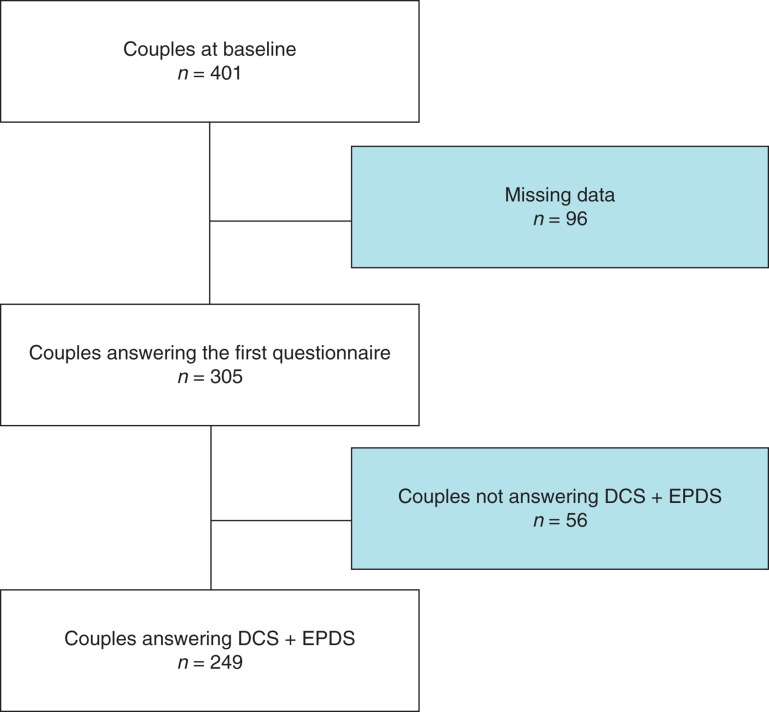
Included couples of the present study.

Due to a printing error in the baseline questionnaire, questions regarding the Dyadic Adjustment Scale (DAS) were not included in about one out of four of the baseline questionnaires, meaning that 96 couples had to be omitted from the data set. Of the remaining participants, 32 of the couples did not answer both questionnaires. The sample used in the present study thus consisted of 305 couples (Figure 1).

### Ethical considerations

Ethical permission to conduct the study was granted by the Central Research Ethics Committee of Stockholm.

## Measures

### Dyadic Consensus Subscale (DCS)

DCS is a part of the Dyadic Adjustment Scale (DAS), which is a comprehensive instrument intended to assess relationship quality and evaluate possible needs for therapy according to four subscales: Dyadic Consensus, Dyadic Satisfaction, Dyadic Cohesion, and Affectional Expression (26). DAS can be used by women as well as men (28) and permits comparisons of partners' scores and assessments of individuals in married and unmarried cohabiting couples (29). The subscales can be used alone, as in the present study, without losing confidence in the reliability of the measure (26,30).

The DCS measures agreement between partners on matters important to their relationship using 13 self-report questions (Table I) scored on a six-point Likert-type scale ranging from ‘Always disagree’ (scored 0) to ‘Always agree’ (scored 5), resulting in a maximum of 65 points (26). The higher the scores, the more the mother/father thinks that she/he and her/his spouse are agreeing; the lower, the more they perceive that they are disagreeing. The DAS was translated using a conventional test analysis and validated for Swedish conditions (31). The DCS has been found to have a high validity and reliability, with a Cronbach's alpha of 0.84 (31).

**Table I.** Dyadic Consensus Subscale (DCS) presenting differences between mothers and fathers using the Wilcoxon signed rank test. The instruction in the questionnaire read: ‘Most people have disagreements in their relationship. Please make an assessment of the extent to which you and your partner agree or disagree’.

Item content	Positive ranks (Father's score >Mother's score) *n* (%)	Negative ranks (Father's score <Mother's score) *n* (%)	Ties (Father's score = Mother's score) *n* (%)	Mean positive ranks	Mean negative ranks	*P* values
Handling finances	57 (19.4)	44 (15.0)	193 (65.6)	50.85	51.19	0.228
Recreational activities	52 (17.8)	77 (26.4)	163 (55.8)	63.48	66.03	0.024*
Religion	43 (15.4)	46 (16.5)	190 (68.1)	46.73	43.38	0.975
Friends	44 (15.0)	90 (30.6)	160 (54.4)	65.91	68.25	< 0.001***
Conventions (correct or proper behaviour)	58 (20.9)	58 (20.9)	161 (58.1)	61.91	55.09	0.549
Philosophy of life	62 (21.5)	75 (26.0)	152 (52.6)	70.18	68.03	0.372
Socializing with family and friends	58 (19.9)	67 (23.0)	166 (57.0)	61.41	64.37	0.312
Aims and life goals	53 (18.4)	86 (29.9)	149 (51.7)	69.51	70.30	0.006**
Time together	48 (16.6)	78 (26.9)	164 (56.6)	65.56	62.23	0.024*
Important decisions	47 (16.0)	70 (23.9)	176 (60.1)	60.77	57.81	0.071
Household tasks	83 (28.6)	54 (18.6)	153 (52.8)	70.83	66.19	0.007**
Leisure time interests and activities	57 (19.5)	81 (27.7)	154 (52.7)	68.98	69.86	0.045*
Decisions regarding career/personal development	51 (17.5)	86 (29.6)	154 (52.9)	65.67	70.98	0.001**
Total DCS score	101 (39.8)	113 (44.5)	40 (15.7)	99.98	114.22	0.121

Note: Significant *p*-values are bold-faced.

* *p* < 0.05;

** *p* < 0.01;

*** *p* < 0.001.*

### Edinburgh Postnatal Depression Scale (EPDS)

EPDS is developed with the purpose of creating a self-reported questionnaire to screen for postpartum depressive symptoms in women (27). The scale has been translated into several languages, including Swedish (32–34), and validated internationally on mothers (34,35) and fathers (36). The scale has been found to be a useful screening instrument for identifying postpartum depressive symptoms in primary health care (35,37,38). In a systematic review of the EPDS, the sensitivity was found to range from 59% to 100% and specificity from 44% to 97%; positive likelihood ratios ranged from 1.81 to 88 (39).

EPDS uses a ten-item four-point scale with scores ranging between 0 and 3 on each item and a total score of between 0 and 30; the higher the score, the more depressive symptoms. To evaluate an individual's level of postpartum depressive symptoms, the person has to answer all the questions in the EPDS questionnaire. Cox et al. (22,27) recommend a cut-off of >9 to identify the risk of postpartum depression and a cut-off of >11 to identify depressive illness of varying severity (27). Even a relatively mild depression can have long-lasting consequences for the mother–infant relationship (10,34). In the present study, the cut-off of >9 was used for both the mothers and the fathers.

### Statistical analyses

DCS and EPDS are measured on an ordinal scale and are thus primarily analysed using non-parametric methods. Consequently, the values for DCS and EPDS are presented as medians and quartiles, median (q_1_; q_3_). Values for categorical variables are given as frequencies and percentages, *n* (%). The mother and the father in a couple are considered dependent, and all analyses involving comparisons between mothers and fathers are thus analysed using statistical methods for paired data.

McNemar's test was used for comparing the occurrence of postpartum depressive symptoms between mothers and fathers. The correlations between DCS scores, EPDS scores, and between DCS and EPDS scores were calculated with Spearman's rank correlation method. Pearson's chi-square test and Fisher's exact test were used for comparing categorical variables.

The Wilcoxon signed rank test was used for measuring differences between mothers' and fathers' perceived levels of marital discord. The Mann–Whitney *U* test was used for analysing differences regarding the perceived level of relationship discord between mothers with and without postpartum depressive symptoms, as well as between fathers with and without postpartum depressive symptoms. SPSS 17.0 was used for all calculations, and a two-sided *P* value of <0.05 was considered statistically significant.

## Results

### Prevalence of perceived relationship discord

There was no overall disagreement between mothers and fathers in the perceived level of relationship discord (total DCS scores). The median (q_1_; q_3_) total DCS score was 54 (51; 58) for mothers and 53 (51; 57) for fathers, with the correlation between the total DCS scores of mothers and fathers being 0.595 (*P* < 0.001).

Regarding the separate items of the DCS, it was found that the couples disagreed about the perceived level of discord in 7 of the 13 items. These were: *Recreational activities*, *Friends*, *Aims and life goals*, *Time together*, *Household tasks*, *Leisure time interests and activities*, and *Decisions regarding career/personal development.* For all of these items, except *Household tasks*, the number of couples in which the mothers had a higher estimated level of agreement than the fathers was larger than the number of couples where it was the other way around (Table I).

### Prevalence of postpartum depressive symptoms

Among the 305 couples included in the study, 260 (85.2%) of the mothers and 252 (82.6%) of the fathers had answered all EPDS questions and could thus be evaluated for postpartum depressive symptoms. The results showed that 43 (16.5%) of the 260 mothers and 22 (8.7%) of the 252 fathers suffered from postpartum depressive symptoms according to the EPDS cut-off of >9. The median (q_1_; q_3_) total EPDS score was 4 (2; 7) for mothers and 3 (1; 6) for fathers.

Both parents had answered all EPDS questions in 249 (81.6%) of the 305 couples. For these cases, it was possible to compare the occurrence of postpartum depressive symptoms between spouses in a couple. In total, both parents had depressive symptoms in 6 (2.4%) of the 249 couples, and neither of the parents showed any depressive symptoms in 192 (77.1%) of the 249 couples. The father, but not the mother, had depressive symptoms in 15 (6.0%) of the 249 couples, while the mother but not the father had depressive symptoms in 36 (14.5%) of the 249 couples. Compared with the fathers, the mothers more often had depressive symptoms (*P* = 0.005). The correlation between the total EPDS scores of mothers and fathers was 0.287 (*P* < 0.001).

There were no differences between mothers and fathers with, and without, depressive symptoms regarding age, being married/cohabiting with the child's other parent, if the child was first-born, or education level (Table II).

**Table II.** Age, first child, and education level among mothers and fathers with and without depressive symptoms.

	Mothers			Fathers		
	With depressive symptoms	Without depressive symptoms	*P* value	With depressive symptoms	Without depressive symptoms	*P* value
Mean age, years (SD)	30.6 (4.46)	29.9 (5.03)	0.393	32.5 (5.15)	33.0 (5.65)	0.666
First child	45.2%	41.9%	0.692	40.9%	44.7%	0.730
Comprehensive school, 9 y	7.1%	5.1%	0.789	0%	5.3%	0.167
High school, <12 y	61.9%	59.9%		90.9%	73%	
University, ≥12 y	31.0%	35.0%		9.1%	21.7%	

### Association between perceived relationship discord and postpartum depressive symptoms

Mothers and fathers with depressive symptoms scored higher levels of discord compared to parents without depressive symptoms. The median (q_1_; q_3_) total DCS score was 53 (48; 56.5) for mothers with depressive symptoms and 55 (52; 59) for mothers without depressive symptoms. For fathers with depressive symptoms the total DCS score was 49 (44.5; 53) and for fathers without depressive symptoms 54 (51; 58). The correlations between the total DCS and EPDS scores were –0.253 (*P* < 0.001) for mothers and –0.313 (*P* < 0.001) for fathers.

The perceived level of relationship discord was higher for mothers and fathers with self-reported depressive symptoms compared to the parents without depressive symptoms for the total score and regarding the items *Socializing with family and friends*, *Important decisions*, and *Household tasks*. Furthermore, mothers with self-reported depressive symptoms scored higher discord compared to the mothers without depressive symptoms and all fathers concerning the items *Friends* and *Philosophy of life*. However, fathers with self-reported depressive symptoms perceived higher level of discord compared to the fathers without depressive symptoms and all mothers, regarding the items *Recreational activities*, *Time together*, *Leisure time interests and activities*, and *Decisions regarding career/personal development* (Table III).

**Table III.** Relationship discord for mothers and fathers with and without depressive symptoms, calculated with the Mann–Whitney test. EPDS cut-off of >9.

	Mothers			Fathers		
	With depressive symptoms	Without depressive symptoms		With depressive symptoms	Without depressive symptoms	
Item content	Mean rank	Mean rank	*P* value	Mean rank	Mean rank	*P* value
Handling finances	121.32	130.46	0.380	102.64	128.78	0.059
Recreational activities	110.90	131.28	0.065	93.14	129.69	0.009**
Religion	112.24	127.51	0.108	121.45	122.59	0.929
Friends	101.07	134.30	0.003**	109.23	128.15	0.182
Conventions (correct or proper behaviour)	111.84	129.93	0.096	104.83	123.54	0.192
Philosophy of life	100.20	133.33	0.002**	109.36	127.06	0.229
Socializing with family and friends	106.32	132.73	0.017*	98.30	128.66	0.035*
Aims and life goals	112.09	131.63	0.092	111.48	125.77	0.322
Time together	115.57	130.97	0.170	83.59	129.54	0.001**
Important decisions	108.77	132.84	0.032*	88.59	130.13	0.004**
Household tasks	108.46	132.32	0.034*	84.71	129.24	0.002**
Leisure time interests and activities	114.24	131.22	0.124	82.86	130.67	0.001**
Decisions regarding career/personal development	114.23	131.22	0.135	99.93	127.97	0.048*
Total DCS score	92.73	127.37	0.004**	67.74	118.79	0.002**

Note: Significant *p*-values are bold-faced.

* *p* < 0.05;

** *p* < 0.01;

*** *p* < 0.001.*

## Discussion

The findings indicated differences in perceived levels of discord between mothers and fathers regarding the issues: *Recreational activities*, *Friends*, *Aims and life goals*, *Time together*, *Household tasks*, *Leisure time interests and activities*, and *Decisions regarding career/personal development*. For all of the items, except *Household tasks*, the fathers estimated that the couple disagreed more than the mothers did. An explanation may be that mothers actually expect to do more chores than fathers (40). Another explanation might be that the father thinks that the spouse is more annoyed about the household than she really is. This demands knowledge about issues where parents disagree and about how to encourage the couples to communicate about those issues. An interesting finding was that the parents did not perceive that they disagreed about the issue *Handling finances*, while previous research has concluded that disagreements about finances are a major source of marital conflicts (41).

The results of the present study provide further evidence that postpartum depressive symptoms among both mothers and fathers are common. In our study, 16.5% of the mothers and 8.7% of the fathers self-reported depressive symptoms, which was in accordance with previous studies (4–6). Our results indicated that nearly a quarter (23%) of the children had at least one parent with postpartum depressive symptoms. According to Pinheiro et al. at least one parent experienced depressive symptoms in 29% of the couples studied (18). For a young child, to live with one or both parents suffering from depressive symptoms can have a negative effect on the parent–infant interaction and the child's behavioural and vocabulary development (12–14).

In the present study, there was a correlation between marital discord and perceived depressive symptoms. This was consistent with previous research which found that higher levels of depressive symptoms were correlated with lower levels of marital satisfaction (25). In another study, men whose partners suffered from postpartum psychiatric disorders reported greater marital dissatisfaction, and women who perceived satisfying marital relations were less likely to exhibit mental health problems during and after pregnancy (42). The correlation between lower perceived discord and depressive symptoms was stronger for the fathers than for the mothers. One explanation for this might be that women often have larger social networks than men (43). The connection between a high perceived level of discord and depressive symptoms may partly be due to the fact that if the mother or father feels unhappy it is easy to blame the partner (44).

There was a correlation between higher levels of discord among mothers and fathers for the items: *Socializing with family and friends*, *Important decisions*, and *Household tasks* and perceived and depressive symptoms. If a person feels down it might be easy to think that her/his spouse does not understand the issues they have. For mothers, there was a correlation between higher levels of discord for the items: *Friends* and *Philosophy* and depressive symptoms. One explanation for this could be that a mother with depressive symptoms requires support from the father and wants him to prioritize herself and their child, as opposed to their friends.

A correlation existed between higher levels of discord among fathers for the items: *Recreational activities*, *Time together*, *Leisure time interests and activities*, and *Decisions regarding career/personal* and depressive symptoms. One could assume that some fathers feel that their spouses do not have enough time to spend with them. Another reason may be that the father misses time for himself after the childbirth. Some recent fathers do not have a clear idea of what it means to be a father and might need support to assume their new role (45). If we had investigated marital discord after several weeks/months, the parents might have given different responses.

### Limitations and strengths

The present study has a number of limitations that must be acknowledged. Firstly, DCS and EPDS were not measured on the same occasion so we do not know if the depressive symptoms were present at the time the marital consensus was assessed and vice versa. Another limitation was that marital discord was assessed 1 week after childbirth, i.e. a period in life that includes an overwhelming experience for most couples. However, the reason for this decision was that we intended to create a baseline variable for comparison with the 3-month assessments. Another option would have been to measure marital discord during pregnancy, although this is a period in life when many couples experience other types of problems (46).

EPDS has only occasionally been validated on men (36), and, to our knowledge, there has been no validation on men in Sweden, which is a shortcoming. The present study's EPDS cut-off of >9 increases the rate of false-positive postpartum depressions compared to a cut-off of >12. The latter gives few false-positives, but the sensitivity is far from adequate (47) and will miss a considerable number of cases (39). A cut-off of >9 is, however, recommended for routine examinations when the scale is used by primary care workers (27). Choosing the cut-off of >9, which has been used in other Swedish studies (48–50), also gives study groups large enough to compare the mothers' and fathers' DCS and EPDS scores.

Another limitation of the present study was that the printing error in the baseline questionnaire, with about one out of four questionnaires missing the DAS questions, resulted in 96 couples not being possible to analyse, and they were thus removed from the data set. However, the missing questionnaires were scattered across the field meaning that couples with complete questionnaires could be found in all communities.

The major strength of the present study was that both parents of the child were included which facilitated a description and comparison of their DCS and EPDS scores with each other. However, we could not control whether the couples influenced each other when filling in the questionnaires, even though our intention was, and the instructions emphasized, that the parents should fill in the questionnaires separately.

### Suggestions for clinical implications

A recommendation that can be based on our results is that professionals in antenatal care, child health centres, as well as family caregivers should be conscious that mothers and fathers may have different views on relationship consensus, and that perceived discord can be related to postpartum depressive symptoms. The results from the present study might help professionals to make parents aware that they sometimes believe that their spouses are disagreeing with them, while, in reality, they are not. The professionals should also be aware of the high level of depressive symptoms in women and men who have recently become parents in order to minimize the harmful effects for the individual, the relationship, and the child. Further research is needed to examine perceived relationship discord and the development of depressive symptoms over a longer term.
